# Cell-free DNA concentration and fragment size as a biomarker for prostate cancer

**DOI:** 10.1038/s41598-021-84507-z

**Published:** 2021-03-03

**Authors:** Emmalyn Chen, Clinton L. Cario, Lancelote Leong, Karen Lopez, César P. Márquez, Carissa Chu, Patricia S. Li, Erica Oropeza, Imelda Tenggara, Janet Cowan, Jeffry P. Simko, June M. Chan, Terence Friedlander, Alexander W. Wyatt, Rahul Aggarwal, Pamela L. Paris, Peter R. Carroll, Felix Feng, John S. Witte

**Affiliations:** 1 Department of Epidemiology and Biostatistics, University of California, San Francisco, CA USA; 2 Department of Urology, University of California, San Francisco, CA USA; 3 Department of Anatomic Pathology, University of California, San Francisco, CA USA; 4 Division of Hematology/Oncology, University of California, San Francisco, CA USA; 5 School of Medicine, Stanford University, Stanford, CA USA; 6 Vancouver Prostate Centre, Department of Urologic Sciences, University of British Columbia, Vancouver, BC Canada; 7 Department of Radiation Oncology, University of California, San Francisco, CA USA

**Keywords:** Tumour biomarkers, Urological cancer

## Abstract

Prostate cancer is the most commonly diagnosed neoplasm in American men. Although existing biomarkers may detect localized prostate cancer, additional strategies are necessary for improving detection and identifying aggressive disease that may require further intervention. One promising, minimally invasive biomarker is cell-free DNA (cfDNA), which consist of short DNA fragments released into circulation by dying or lysed cells that may reflect underlying cancer. Here we investigated whether differences in cfDNA concentration and cfDNA fragment size could improve the sensitivity for detecting more advanced and aggressive prostate cancer. This study included 268 individuals: 34 healthy controls, 112 men with localized prostate cancer who underwent radical prostatectomy (RP), and 122 men with metastatic castration-resistant prostate cancer (mCRPC). Plasma cfDNA concentration and fragment size were quantified with the Qubit 3.0 and the 2100 Bioanalyzer. The potential relationship between cfDNA concentration or fragment size and localized or mCRPC prostate cancer was evaluated with descriptive statistics, logistic regression, and area under the curve analysis with cross-validation. Plasma cfDNA concentrations were elevated in mCRPC patients in comparison to localized disease (OR_5ng/mL_ = 1.34, P = 0.027) or to being a control (OR_5ng/mL_ = 1.69, P = 0.034). Decreased average fragment size was associated with an increased risk of localized disease compared to controls (OR_5bp_ = 0.77, P = 0.0008). This study suggests that while cfDNA concentration can identify mCRPC patients, it is unable to distinguish between healthy individuals and patients with localized prostate cancer. In addition to PSA, average cfDNA fragment size may be an alternative that can differentiate between healthy individuals and those with localized disease, but the low sensitivity and specificity results in an imperfect diagnostic marker. While quantification of cfDNA may provide a quick, cost-effective approach to help guide treatment decisions in advanced disease, its use is limited in the setting of localized prostate cancer.

## Introduction

Prostate cancer accounts for approximately 20% of all new cancer diagnoses in American men. While individuals diagnosed with localized disease have a 98% 5-year survival rate, an estimated 33,330 men will die from aggressive and metastatic disease in 2020^1^. There are a number of existing biomarkers routinely used for prostate cancer diagnosis and monitoring, including prostate-specific antigen (PSA), PHI, 4Kscore, PCA3 expression, parametric MRI, and hypermethylation of GSTP1, APC, and RASSF1^2,3^. These have varying levels of sensitivity and specificity, and additional biomarkers for prostate cancer are necessary to reduce over-diagnosis and over-treatment of this common, but complex disease.

Cell-free DNA (cfDNA) is a promising, minimally invasive biomarker that may originate from cell lysis, apoptosis, necrosis, and active release of DNA fragments into circulation^4–7^. In healthy individuals, cfDNA is predominantly of hematopoietic origin^8^. In cancer patients, cfDNA includes DNA of hematopoietic origin, as well as circulating tumor DNA (ctDNA) derived from tumor cells. Two commonly used methods to profile cfDNA are: (1) quantification of cfDNA based on spectrophotometry, electrophoresis, or quantitative PCR (qPCR); and (2) genomic interrogation of ctDNA fragments with next-generation sequencing, BEAMing (beads, emulsion, amplification, and magnetics), or droplet digital PCR (ddPCR).

While genomic interrogation allows for the detection of cancer-specific fragments, this can have a number of challenges (e.g., sufficient cfDNA, sequencing depth, and mutation panel selection). In contrast, quantification of overall cfDNA concentrations and assessment of cfDNA fragment size may provide a quick, cost-effective method in addition to other biomarkers such as PSA, and can deliver insight into whether a patient should undergo further biopsy and potentially genomic testing.

Elevated concentrations of cfDNA were initially reported in patients with leukemia and autoimmune disease^9,10^. Subsequent studies have also determined that high concentrations of cfDNA are typically associated with poor survival in several cancers^11,12^. For prostate cancer, increased plasma cfDNA concentrations were found in patients with lymph node and distant metastases^13^. Elevated preoperative serum cfDNA concentrations in men with localized prostate cancer who underwent RP have been associated with PSA recurrence, independent of surgical margin and lymph node status, as well as Gleason score and pathologic stage^14^.

In addition to overall cfDNA concentrations, cfDNA fragment size may provide diagnostic and prognostic value. DNA integrity, which measures the ratio of all cfDNA fragments (ALU 247 bp) to shorter fragments (ALU 115 bp) has distinguished prostate cancer from benign prostatic hyperplasia (BPH)^15^. Pre-treatment cfDNA concentration and fragment size were predictive of advanced pancreatic cancer progression-free survival and overall survival^16^. Furthermore, tumor fragments in cfDNA appeared shorter in size than fragments that originated from non-malignant cells^17–19^.

Here, we evaluate whether baseline plasma cfDNA concentrations and cfDNA fragment size can differentiate among: (1) men with prostate cancer and controls; and (2) clinical characteristics or biochemical recurrence among men with localized disease (i.e., PSA at diagnosis, Gleason, organ confinement, extraprostatic extension, seminal vesicle invasion, lymph node invasion, and RNA gene expression). While cfDNA concentration data was available for mCRPC and localized prostate cancer groups, cfDNA fragment size data was only available for patients with localized disease.

## Materials and methods

### Patient cohort

From August 2015 to November 2019, biological samples from a total of 268 individuals were included in this study: 34 healthy donors, 112 patients with localized prostate cancer, and 124 mCRPC patients (Table 1). Twenty-eight healthy donor samples were obtained from StemCell (StemCell Technologies, Seattle, WA), and six healthy samples were collected from volunteers at UCSF. All patients with localized disease underwent radical prostatectomy (RP) at UCSF, and 35/112 patients had a Decipher score of RNA gene expression available (GenomeDx Biosciences, Vancouver, British Columbia, Canada)^20^. For the patients with localized disease, blood samples were collected the day of surgery before RP, and for five of these men blood samples were collected two months after surgery. Clinicopathologic variables that play an important role in surgical management after prostatectomy were also collected, including clinical T stage, pathologic Gleason score, preoperative PSA, and risk prediction models including the Cancer of the Prostate Risk Assessment Postsurgical (CAPRA-S) score and the Decipher score^21,22^. Known predictors of biochemical recurrence (BCR), including organ confinement, extraprostatic extension, seminal vesicle invasion, and lymph node involvement were also identified^23^. Biochemical recurrence was defined as two consecutive PSA levels of ≥ 0.2 ng/mL eight weeks after surgery.

**Table 1:** Clinical characteristics of individuals included in the study at baseline.

	Healthy N = 34	Localized N = 112	mCRPC N = 122
**Age (years)**			
Median ± IQR	60 ± 17	65 ± 10	71 ± 9
Range	41–74	43–78	47–91
Pathologic Gleason*			
6	–	6	–
7	–	75	–
8–10	–	30	–
**Pathologic stage**			
Organ confined (pT2)	–	46	–
Not organ confined (pT3, pT4)	–	66	–
Extraprostatic extension (pT3a)	–	43	–
Seminal vesicle invasion (pT3b)	–	15	–
Lymph node involvement (N1)	–	17	–
**PSA (ng/mL)**			
Median ± IQR	–	6.9 ± 7.4	50.8 ± 128
Range	–	1.21–70.0	0–3007
**cfDNA concentration (ng/mL)**^†^			
Median ± IQR	7.9 ± 4.0	6.7 ± 5.8	13.8 ± 28.1
Range	0.29–16.9	1.22–53.9	1–1380
**cfDNA fragment size (bp)**			
Median ± IQR	177.5 ± 29.5	173 ± 6	–
Range	142–265	135–280	–

*One man with unknown data in the cohort.

^†^Concentration data collected on a subset of individuals (31 healthy, 45 localized, and 122 mCRPC).

While most of the cohort was collected prospectively, a subset of 110 mCRPC patients were recruited through the Stand Up 2 Cancer/Prostate Cancer Foundation-funded West Coast Prostate Cancer Dream Team Project (IRB 12-10340). Fourteen mCRPC patients were recruited through UCSF^24^. For mCRPC patients, blood samples were collected prior to treatment initiation. Clinicopathologic characteristics were collected for all patients (Table 1). All experimental protocols were approved by Human Research Protection Program Institutional Review Board at University of California, San Francisco (IRB 11-05226 and IRB 12-09659). All subjects gave written informed consent in accordance with the Declaration of Helsinki.

### cfDNA extraction from blood

For healthy controls, whole peripheral blood samples were collected from individuals in PAXgene Blood ccfDNA tubes (Qiagen, Redwood City, CA). Healthy samples collected by StemCell were shipped at room temperature, arriving within 7 days for sample processing. Whole peripheral blood samples were collected immediately before surgery for patients with localized disease or at the time of follow-up and before treatment initiation for mCRPC patients. Plasma was generated from whole blood samples within 2 h for blood collected in K3EDTA tubes or within 7 days for blood collected in PAXgene Blood ccfDNA tubes with a two-step centrifugation protocol: first centrifuging the blood at 1900 *g* for 10 min at 21 °C, followed by centrifugation of the supernatant at 16,000 *g* for 10 min to remove leukocytes and cellular debris. DNA was extracted from 7 to 55 mL of plasma using the Qiagen QIAamp Circulating Nucleic Acid Kit (Qiagen, Redwood City, CA), and double eluted with 40 μL of Qiagen Elution Buffer. Extracted DNA was stored at − 20 °C prior to further analysis.

### cfDNA fragment size and concentration

Extracted DNA was quantified with a Qubit 3.0 fluorometer and a DNA dsDNA HS Assay Kit (Life Technologies, Carlsbad, CA), as well as on the 2100 Bioanalyzer with High Sensitivity DNA Chips (Agilent Technologies, Santa Clara, CA) for assessment of sample purity, concentration, and fragment size distribution according to the manufacturer’s instructions. The average fragment size was determined with the Agilent 2100 Bioanalyzer Expert software, and calculated across the first three peaks 75–675 bp corresponding to the length of nucleosomal footprints and linkers derived from apoptotic cells (Supplementary Figure S1). The final plasma cfDNA concentrations were calculated by adjusting for the initial plasma and final elution volumes, and quantified with a Qubit 3.0 for a subset of patients (Supplementary Table S1). Assessment of cfDNA fragment size and concentration was performed without prior knowledge of clinical data. Average cfDNA fragment size was not available for mCRPC patients, since samples were not available for analysis on the 2100 Bioanalyzer.

### Statistical analysis

Our primary analysis assessed the relationship between cfDNA concentration or average fragment size and prostate cancer, comparing three groups: healthy controls, men with localized disease, and men with mCRPC. Here we used descriptive statistics, logistic regression, and receiver operating characteristic (ROC) curves. Since cfDNA concentration and average fragment size were not normally distributed (P < 0.001, Shapiro–Wilk test), we evaluated the difference in descriptive statistics across prostate cancer diagnoses using the Mann–Whitney non-parametric test. We also evaluated differences in cfDNA concentration quantified between 90 and 150 bp, which is known to be enriched for circulating tumor DNA fragments specifically^17^. Then, we further investigated the potential relationship between cfDNA concentration and prostate cancer diagnoses using logistic regression models (crude, and then adjusting for age at time of blood draw and baseline PSA when available). However, additional clinicopathological variables were not available for mCRPC patients. The ability of cfDNA concentration to discriminate between prostate cancer diagnoses was further assessed based on the area under the curve (AUC) from a Receiver Operating Characeristics (ROC) curve analysis with k-fold cross-validation (a minimum of ten observations per fold) and bootstrap resampling (n = 100). These analyses were also performed with average cfDNA fragment size to distinguish patients with localized disease from controls, but not assessed for mCRPC patients since their samples were not available for analysis on the 2100 Bioanalyzer.

We also undertook secondary analyses investigating the relationship between baseline cfDNA concentration or fragment size and clinical characteristics among patients with localized disease. For continuous characteristics, comparisons were made using cfDNA concentration and Pearson correlation coefficients (i.e., age at diagnosis, PSA at diagnosis, Decipher score, time to salvage therapy, average cfDNA fragment size, and postoperative CAPRA-S score). For categorical clinical features, we assessed the potential relationship between log-transformed cfDNA concentration (for normality) and other clinical features (pathologic Gleason score, organ confinement, extraprostatic extension, seminal vesicle invasion, pathologic lymph node status, biochemical recurrence, and clinical T stage) with Student’s t–tests. Mann–Whitney U-tests were used to assess the association between the average cfDNA fragment size and the same clinical features. As with localized disease, we also evaluated the relationship between cfDNA concentration and age at time of blood draw for healthy individuals and mCRPC patients. Finally, we evaluated the association between cfDNA concentration or fragment size and biochemical recurrence-free survival with Cox proportional hazards models for patients with localized disease. All data analyses were performed using R version 3.6.1^25^.

## Results

### cfDNA concentration and prostate cancer

**Figure 1:**
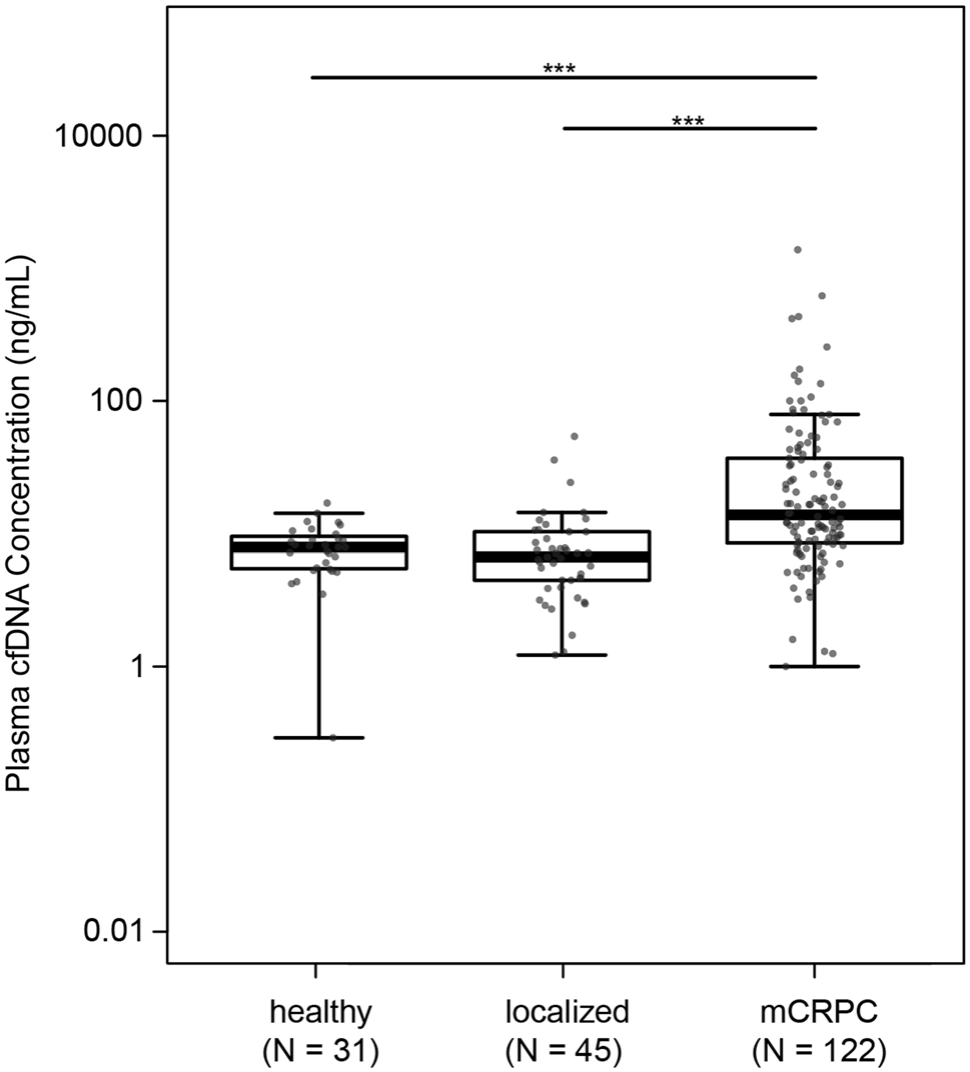
Distribution of plasma cfDNA concentration in healthy individuals, patients with localized disease, and patients with mCRPC. Boxplots and points identify the minimum, interquartile range, median, and maximum values for each group. The Mann–Whitney test was applied to test differences in cfDNA concentration between groups. *** P < 0.0001.

The median cfDNA concentration was 7.9 ng/mL (IQR, 4.0 ng/mL) for controls, 6.7 ng/mL (IQR, 5.8 ng/mL) for patients with localized disease, and 13.8 ng/mL (IQR, 28.1 ng/mL) for patients with mCRPC (Table 1; Fig. 1). The average cfDNA levels in mCRPC patients were statistically significantly higher than those observed in controls (P < 0.0001) or those with localized prostate cancer (P < 0.0001).

These observations were further supported by results from the logistic regression models, including those adjusting for age and PSA levels (Table 2). A 5 ng/mL increase in cfDNA concentration was positively associated with mCRPC in comparison to localized disease (OR_crude_ = 1.47, P = 0.0017; OR_adjusted_ = 1.34, P = 0.027) or to being healthy (OR_crude_ = 1.93, P = 0.0025; OR_adjusted_ = 1.69, P = 0.034). Plasma cfDNA concentration was not associated with having localized disease in comparison to healthy individuals (OR_crude_ = 1.10, P = 0.64; OR_adjusted_ = 1.05, P = 0.72).

**Table 2:** Association between increase in cfDNA concentration (5 ng/mL) or in cfDNA fragment size (5 bp) and prostate cancer status. Results from crude univariate and adjusted multivariate logistic regression analyses (adjusted for age, and PSA when available).

cfDNA measure	Prostate cancer status	Variable	Crude			Adjusted	
			Odds ratio (95% CI)		P-value	Odds ratio (95% CI)	P-value
cfDNA concentration (5 ng/mL)	Localized vs healthy	cfDNA concentration	1.10 (0.82–1.61)		0.64	1.05 (0.77–1.69)*	0.72
		Age	–		–	1.54 (1.10–2.10)	0.01
	mCRPC vs healthy	cfDNA concentration	1.93 (1.34–3.18)		0.0025	1.69 (1.16–2.93)*	0.034
		Age	–		–	2.39 (1.69–3.43)	1E–06
	mCRPC vs localized	cfDNA concentration	1.47 (1.22–2.01)		0.0017	1.34 (1.05–1.76)^†^	0.027
		Age	–		–	1.76 (1.28–2.60)	0.002
		PSA	–		–	1.54 (1.22–2.01)	0.0008
cfDNA fragment size (5 bp)	Localized vs healthy	cfDNA Fragment size	0.86 (0.73–0.9)		0.003	0.77 (0.66–0.90)*	0.0008
		Age	–		–	1.61 (1.22–2.10)	0.001

*Adjusted for age.

^†^Adjusted for age and PSA.

**Figure 2:**
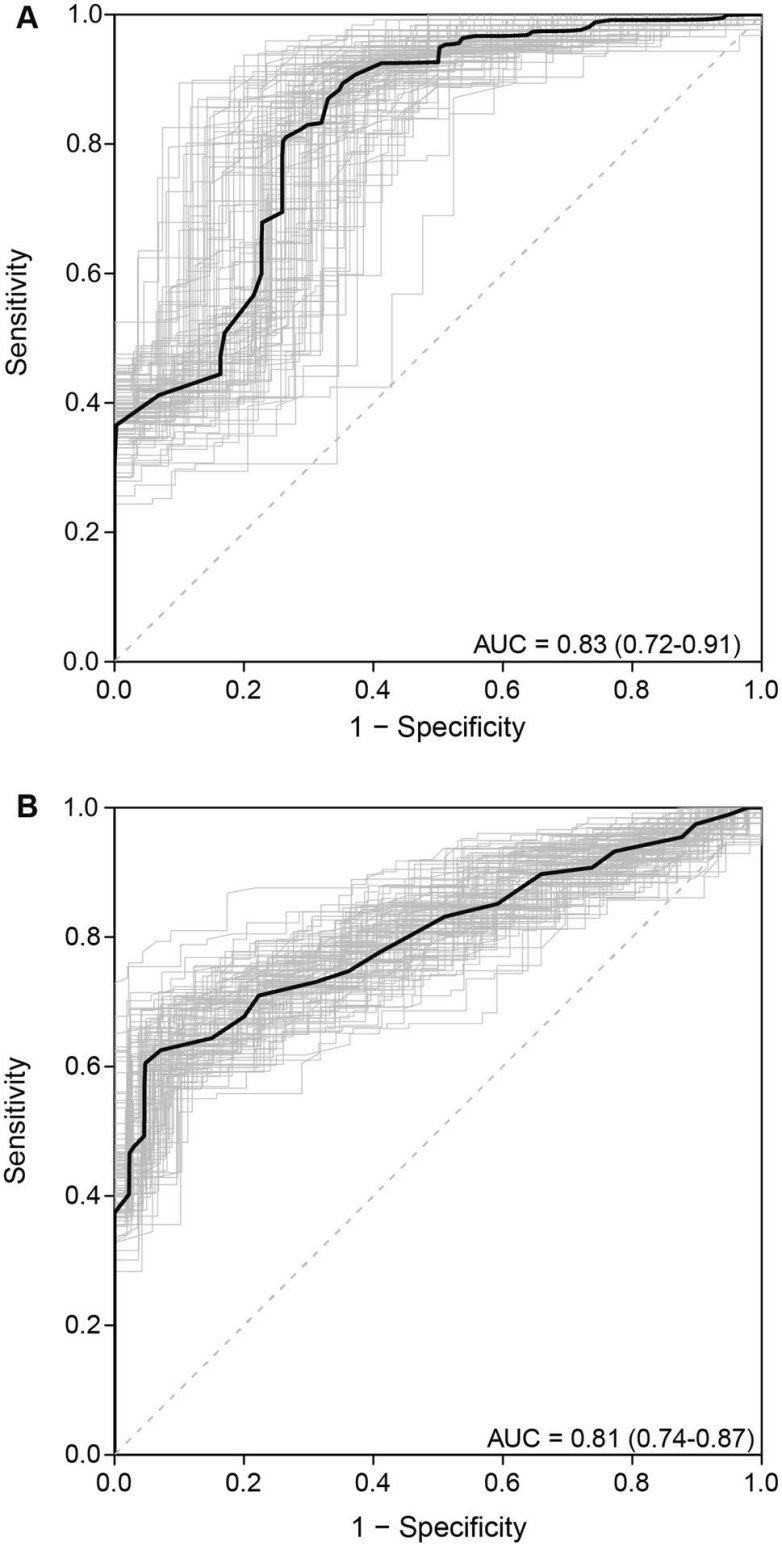
Receiver operating characteristic (ROC) curves for cfDNA concentration comparison between **(A)** healthy individuals and mCRPC, and **(B)** patients with localized disease and mCRPC. Area under the curve (AUC) and 95% CI were estimated with k-fold cross-validation and bootstrap resampling.

In our ROC curve analysis, plasma cfDNA concentration was able to distinguish between mCRPC patients from healthy individuals and those with localized disease (Fig. 2), with an estimated AUC of 0.83 (95% CI, 0.72–0.91) and 0.81 (95% CI, 0.74–0.87), respectively.

### cfDNA fragment size and prostate cancer

**Figure 3:**
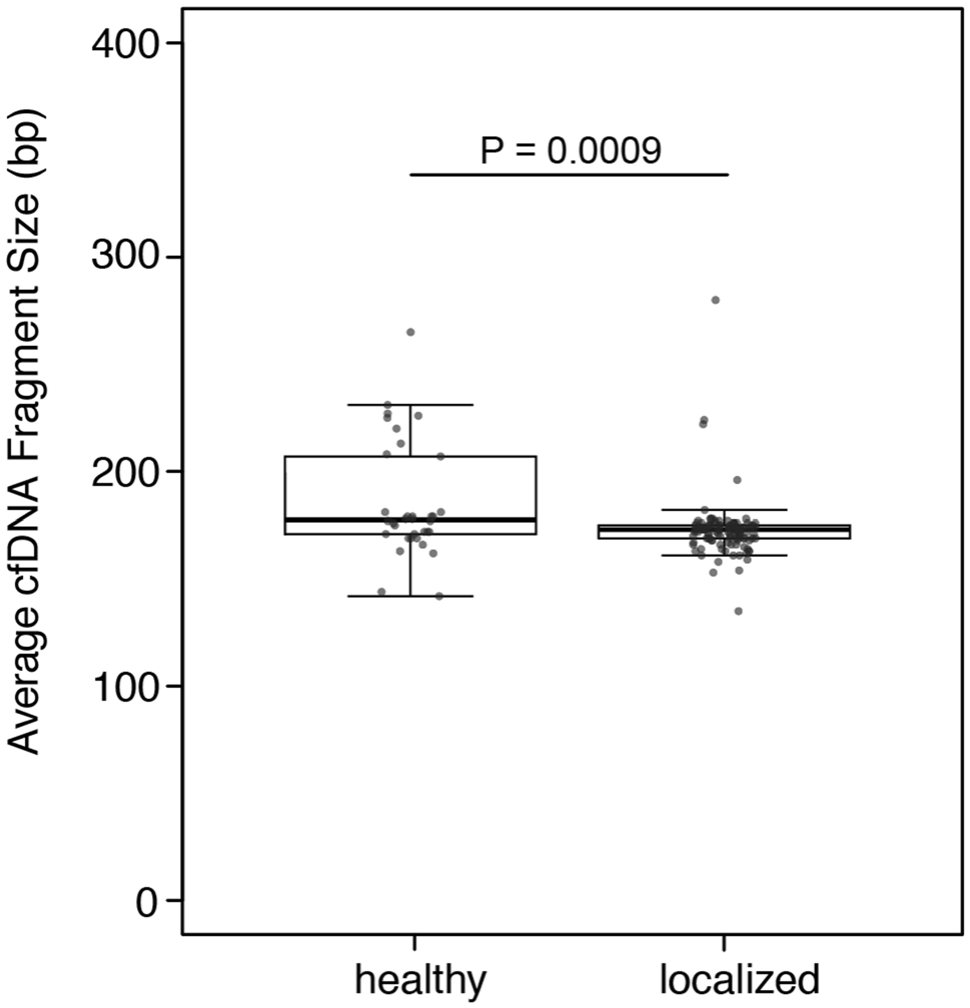
Distribution of average fragment size in healthy individuals and patients with localized disease (P = 0.0009). Boxplots and points identify the minimum, interquartile range, median, and maximum values for each group. A Mann–Whitney U-test was performed to test the difference in cfDNA fragment size.

The median of the average cfDNA fragment size in patients with localized disease was 173 bp (range, 135–280 bp), and in controls was 177.5 bp (range, 142–265 bp) (Table 1). This lower average cfDNA fragment size in patients with localized disease was statistically significantly different from that observed in controls (P = 0.0009, Fig. 3). Results from the logistic regression analysis further indicate that average fragment size was inversely associated with localized prostate cancer (in comparison to healthy individuals): for a 5 bp increase in fragment size, the OR_crude_ = 0.86 (P = 0.003) and OR_adjusted_ = 0.77 (P = 0.0008; Table 2). The estimated ROC AUC for distinguishing between healthy individuals and patients with localized prostate cancer using average cfDNA fragment size was 0.64 as defined by k-fold cross-validation, with 88% specificity and 56% sensitivity at an optimal threshold of 176.5 bp where the sum of sensitivity and specificity are at a maximum. There was no difference in cfDNA concentration quantified across 90–150 bp between healthy individuals and patients with localized disease (Supplementary Figure S4)^17^.

### cfDNA concentration/fragment size and clinical characteristics in localized prostate cancer

There were no statistically significant differences in cfDNA concentration or fragment size for the clinical characteristics/outcomes evaluated here (Supplementary Table S2; Supplementary Table S3). Specifically, cfDNA concentration or fragment size did not appear to substantively differ across: pathologic Gleason score, organ confinement, extraprostatic extension, seminal vesicle invasion, time to biochemical recurrence, average cfDNA fragment size, clinical T stage, pathologic lymph node status, age at diagnosis, PSA at diagnosis, Decipher score, time to salvage therapy, and CAPRA-S score (Supplementary Figure S2; Supplementary Table S2; Supplementary Table S3). Additionally, no clear correlation was observed between cfDNA concentration and age at time of blood draw for healthy individuals, patients with localized disease, or patients with mCRPC (Supplementary Figure S3).

## Discussion

This study found that plasma cfDNA concentration and fragment size may have limited diagnostic and prognostic value for detecting and profiling prostate cancer. Specifically, plasma cfDNA concentrations may help identify patients with advanced disease, but is unable to distinguish patients with early stage disease from healthy individuals. While average cfDNA fragment size may be used to distinguish between these two groups, its low specificity and sensitivity result in poor diagnostic ability.

In the multivariate model that adjusted for age and PSA, plasma cfDNA concentration remained an independent predictor of mCRPC, indicating that cfDNA concentration may capture different biological processes than PSA and provides additional information (Table 2). Average cfDNA fragment size was predictive of localized disease, although with a low AUC indicative of poor discrimination. Looking at follow-up fragment size measures available for five patients, we found that one patient had a shorter average fragment size two months after surgery, and also exhibited post-treatment elevated PSA (Table 2; Supplementary Figure S6). In combination, these findings suggest that quantification of cfDNA overall may have limited utility in identifying prostate cancer patients.

While the biological mechanisms underlying decreased fragment size in cancer patients are not well-understood, differences in nucleosome positioning and DNA methylation may result in varied DNA degradation. Our finding that the overall average fragment size in localized patients was shorter and more fragmented than in healthy individuals is consistent with the findings of studies assessing fragment size in patients with hepatocellular carcinoma and advanced pancreatic cancer^18,26^. The proportion of cfDNA fragments shorter than 150 bp is also increased for multiple cancer types when compared to healthy fragment sizes with shallow genome-wide sequencing^17^. Localized prostate cancers are characterized by initial accumulation of clonal point mutations and deletions, with subsequent branching copy number gains where amplified regions are relatively enriched for tumor DNA, possibly modifying intracellular DNA degradation processes and mechanisms of DNA release and contributing to the size differences that were observed^27^.

The relatively short follow-up time for a protracted disease like prostate cancer is a limitation in this study. Of the 112 patients who underwent surgery, 24 patients experienced biochemical recurrence with a median follow-up time of three years (range, 9–1704 days), and it was not feasible to identify patients with localized disease who may have progressed to metastatic disease. This study did not include patients with metastatic disease who were not resistant to hormone therapy (i.e. castration sensitive), limiting the generalizability of these results to the full spectrum of this disease.

We processed samples in a manner that maximized the quality and quantity of extracted cfDNA^28–32^. An initial low-speed centrifugation step followed by high-speed centrifugation was used to reduce the amount of cellular debris and genomic DNA in the sample. Importantly, there was no significant difference in cfDNA concentration for samples collected in K3EDTA and PAXgene Blood ccfDNA tubes in the localized cohort (Supplementary Figure S5). However, the slightly increased cfDNA concentrations observed in controls may be due to cell lysis during transit, since whole blood was collected from individuals at a donor center in Kent, Washington and shipped overnight to San Francisco, California, whereas the patient samples were collected and processed onsite. Additionally, the lower overall cfDNA concentrations found in the localized cohort may be due to the subduing effect of anesthetic agents on cell death, which were administered prior to blood sample collection before surgery^33^. While data comparison across studies is difficult due to differences in sample collection and processing, most studies demonstrate the diagnostic role of cfDNA^12^. To quantify cfDNA, the Qubit 3.0 and the Agilent 2100 Bioanalyzer were used as a straightforward approach, albeit potentially less accurate than sequencing. In a clinical setting, an affordable, rapid, and straightforward test is critical to minimizing disruption to standard workflows while providing additional information. However, cfDNA quantification is a complementary approach that could help identify patients who may benefit from cfDNA sequencing.

Bastian et al. observed significant associations between cfDNA concentration and clinical characteristics in a cohort of patients with localized disease that experienced biochemical recurrence, supporting the hypothesis that cfDNA quantification may have more utility in the management of more advanced disease^14^. The lack of associations observed between cfDNA concentration and clinical characteristics in our study may be due to differences in the study cohorts. While all patients in the Bastian et al. study experienced BCR, only 24 of 112 patients experienced BCR in our study^14^. Additionally, the subgroup analyses within localized prostate cancer may be limited in its power to detect an effect due its small sample size of forty-five individuals. While the median age of healthy individuals is similar to those of localized prostate cancer patients, further cfDNA studies would benefit from the selection of a larger cohort of age-matched controls.

A previous study demonstrated that in pre-treatment speciments, shorter cfDNA fragment size and elevated cfDNA concentrations were associated with shorter progression-free survival and overall survival in patients with advanced pancreatic cancer^26^. Due to the relatively short follow-up time in this study, future longitudinal studies evaluating disease progression from localized to metastatic disease are necessary to elucidate the value of analyzing cfDNA concentration and fragment size in the context of prostate cancer.

While the exact mechanism of cfDNA release into circulation remains unknown, apoptosis, lysis, necrosis, and active secretion have been identified as potential routes^6,34^. The cfDNA found in healthy individuals originates from hematopoietic cells, and likely reflects the processes of regulated cell turnover in these cells^8^. In patients with cancer, cfDNA includes both DNA fragments from hematopoietic cells, as well as fragments from tumor cells. Future studies evaluating the mechanisms of release will help elucidate the underlying biology of this biomarker, especially in combination with diagnostic and prognostic information over longer periods of time.

## Conclusion

Collectively, our data demonstrate the limited applications of plasma cfDNA concentration and cfDNA fragment size in prostate cancer. Patients with advanced mCRPC had higher cfDNA concentration than men with localized disease or healthy controls, but no differences were seen between patients with localized disease and healthy controls. While those with localized disease had shorter average fragment sizes than controls, the low sensitivity and specificity suggest poor diagnostic ability. Importantly, cfDNA concentration and fragment size remained independent predictors after adjusting for age and PSA. Future studies assessing both cfDNA concentration and fragment size will be necessary to define optimal cutpoints and assess associations with clinically significant prostate cancer in order to clarify the utility of plasma cfDNA in the context of diagnosis, prognosis, and disease monitoring.

## Supplementary Information

Supplementary Dataset.

Supplementary Information.

## Data Availability

The data supporting the conclusions of this study is included in the article and its Supplementary files.
