# Supplementary material for: Cell-free DNA concentration and fragment size as a biomarker for prostate cancer
Source: Sci Rep. 2021 Mar 3;11:5040. doi: 10.1038/s41598-021-84507-z (PMC7930042; doi:10.1038/s41598-021-84507-z)
Supplement: Supplementary file 2 — Supplementary Information. [file 41598_2021_84507_MOESM2_ESM.pdf]

Cell-free DNA concentration and fragment size as a biomarker for prostate cancer

Emmalyn Chen[1,*], Clinton L. Cario[1], Lancelote Leong[1], Karen Lopez[2], César P. Márquez[4,5], Carissa Chu[2], Patricia S. Li[2], Erica Oropeza[2], Imelda Tenggara[2], Janet Cowan[2], Jeffry P. Simko[2,3], June M. Chan[1,2], Terence Friedlander[4], Alexander W. Wyatt[6], Rahul Aggarwal[4], Pamela L. Paris[2], Peter R. Carroll[2], Felix Feng[2,7], and John S. Witte[1,2,*]

[1] Department of Epidemiology and Biostatistics, University of California, San Francisco, California.
[2] Department of Urology, University of California, San Francisco, California.
[3] Department of Anatomic Pathology, University of California, San Francisco, California.
[4] Division of Hematology/Oncology, University of California, San Francisco, California.
[5] School of Medicine, Stanford University, Stanford, California.
[6] Vancouver Prostate Centre, Department of Urologic Sciences, University of British Columbia, British Columbia, Canada.
[7] Department of Radiation Oncology, University of California, San Francisco, California.

* To whom correspondence should be addressed.
John S. Witte
UCSF Helen Diller Research Building
1450 Third Street
San Francisco CA 94143-0794
(415) 502-6882
JWitte@ucsf.edu

Supplementary Tables & Figures

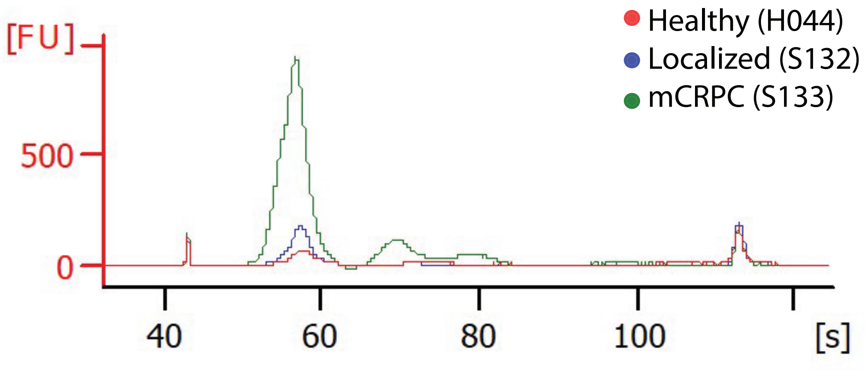

Supplementary Figure S1. Plasma cfDNA concentration and distribution of cfDNA fragment size with representative traces for a healthy individual, patient with localized prostate cancer, and mCRPC patient.

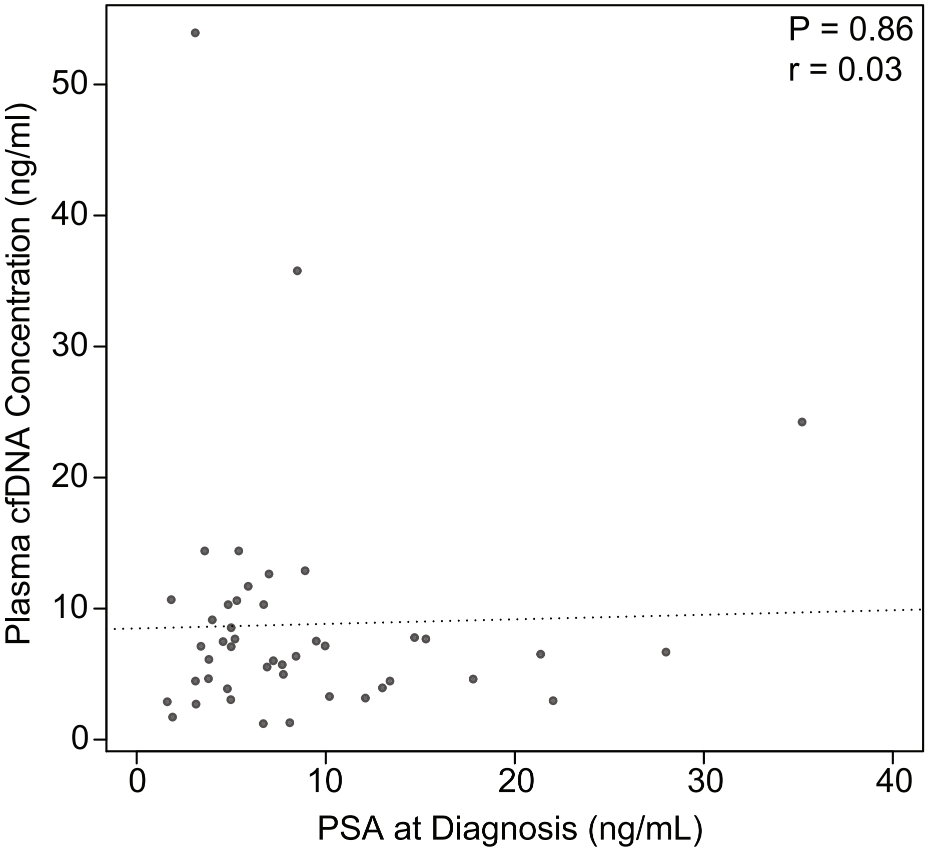

Supplementary Figure S2. Assessment of the relationship between cfDNA concentration and PSA for patients with localized disease (P = 0.86).

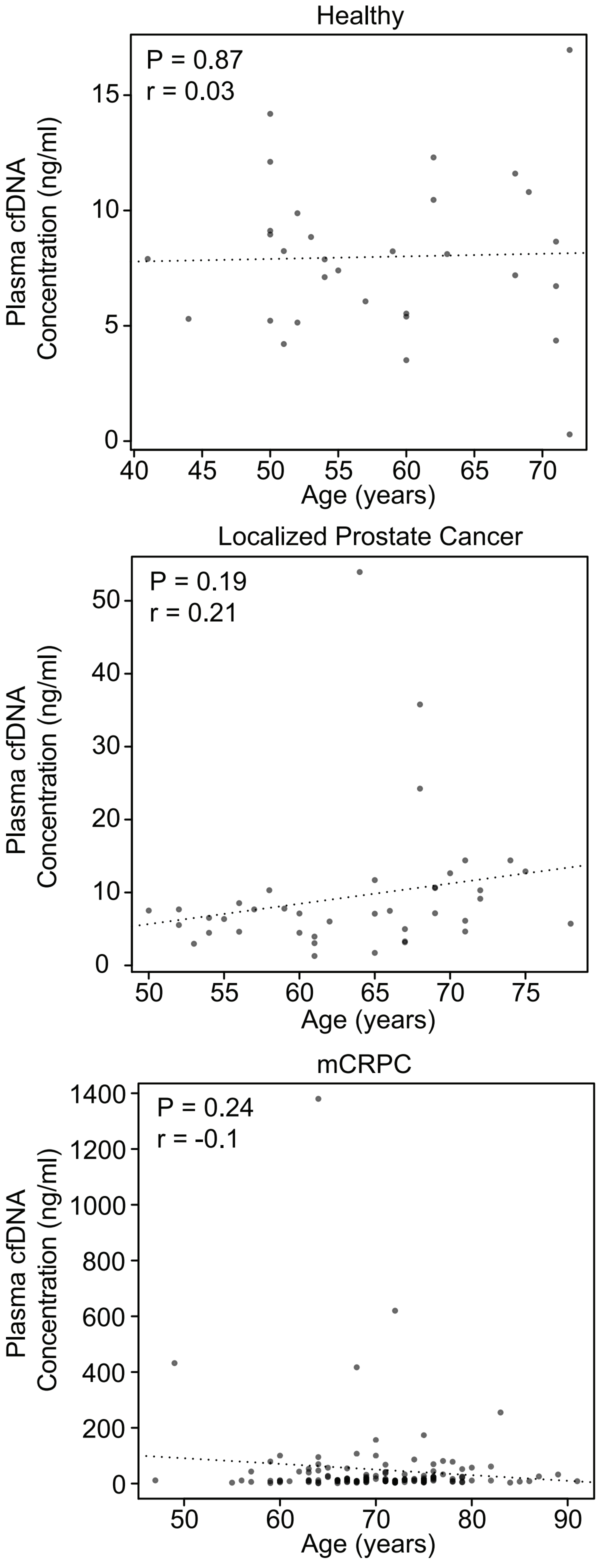

Supplementary Figure S3. Assessment of the relationship between cfDNA concentration and age at time of blood draw.

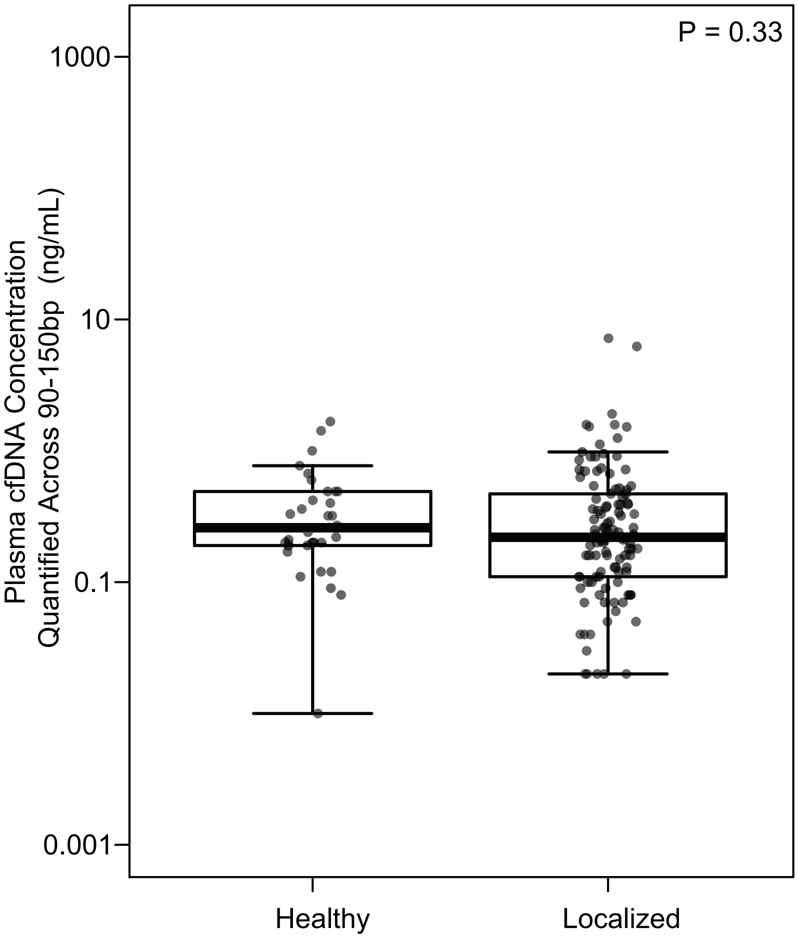

Supplementary Figure S4. Distribution of plasma cfDNA concentration quantified across 90–150bp in healthy individuals and patients with localized disease with the 2100 Bioanalyzer. Boxplots and points identify the minimum, interquartile range, median, and maximum values for each group. A Mann-Whitney U-test was performed to test the difference in cfDNA yields.

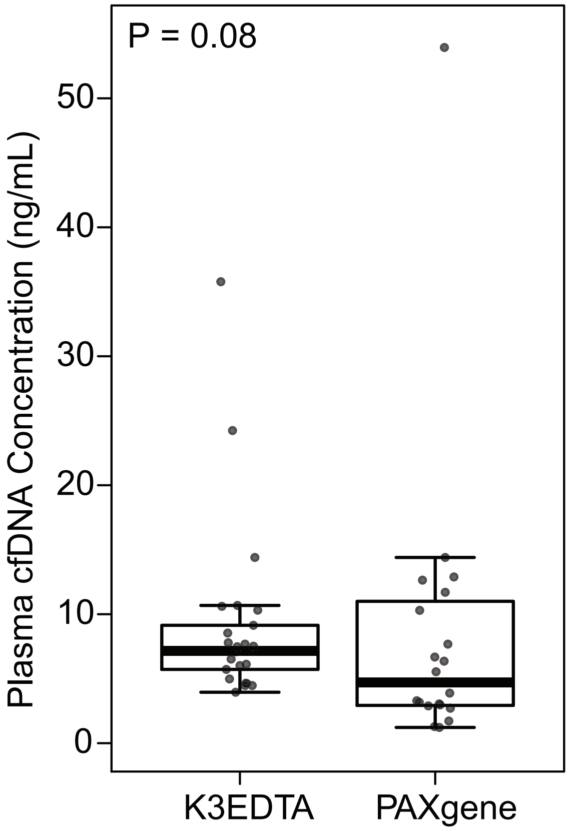

Supplementary Figure S5. Distribution of cfDNA concentration for K3EDTA and Qiagen PAXgene tube types for patients with localized disease.

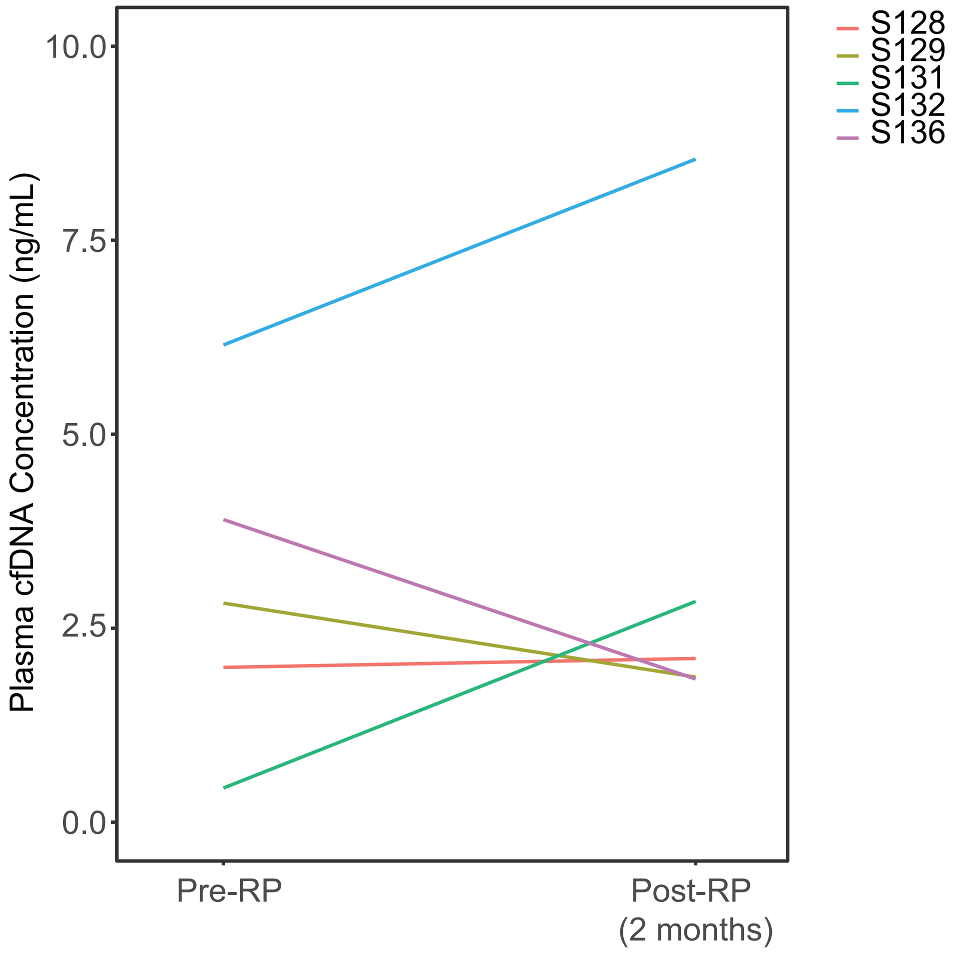

Supplementary Figure S6. Blood samples were collected two months after RP for five patients. Plasma cfDNA concentration increased for two patients (S131 and S132). Patient S131 exhibited a decrease in average cfDNA fragment size from 206bp to 167bp, as well as an elevated PSA of 0.36 ng/mL 64 days after surgery, while the average cfDNA fragment size for patient S132 remained the same at 181bp and PSA levels remained low at 0.03 ng/mL.

Supplementary Table S1. Clinical characteristics of individuals included in the cfDNA concentration analysis.

	Healthy N = 31	Localized N = 45	mCRPC N = 122
**Age (years)**			
Median ± IQR	57 ± 15	65 ± 10	71 ± 9
Range	41 – 72	43 – 78	47 – 91
**Pathologic Gleason***			
6	-	4	-
7	-	28	-
8-10	-	12	-
**Pathologic Stage**			
Organ confined (pT2)	-	24	-
Not organ confined (pT3, pT4)	-	21	-
Extraprostatic extension (pT3a)	-	14	-
Seminal vesicle invasion (pT3b)	-	3	-
Lymph node involvement (N1)	-	5	-
**PSA (ng/mL)**			
Median ± IQR	-	6.7 ± 5.4	50.8 ± 128
Range	-	1.62 – 35.2	0 – 3007
**cfDNA Concentration (ng/mL)**			
Median ± IQR	7.9 ± 4.0	6.7 ± 5.8	13.8 ± 28.1
Range	0.29 – 16.9	1.22 – 53.9	1 – 1380

*One man with unknown data in the cohort.

Supplementary Table S2. Association between log transformed cfDNA concentration or average cfDNA fragment size and categorical clinical features for patients with localized disease.

Clinical Feature	Number of Patients	Average cfDNA Concentration (ng/mL)	Average cfDNA Fragment Size (bp)
Pathologic Gleason score			
≤ 7 (3+4)	25	8.90	169.8
≥ 7 (4+3)	20	8.65	174.7
P		0.19	0.09
Organ confinement			
Confined (pT2)	24	8.25	172.7
Not confined (pT3, pT4)	21	9.40	172.8
P		0.80	0.69
Extraprostatic extension			
Negative (pT2)	24	8.25	172.7
Positive(pT3a)	14	12.33	170.7
P		0.29	0.67
Seminal vesicle invasion			
Negative (pT2, pT3a)	38	9.75	171.7
Positive (pT3b)	3	3.74	171.9
P		0.15	0.45
Pathologic lymph node			
Negative (pN0)	23	8.45	172.2
Positive (pN1)	5	8.49	173.8
P		0.90	0.69
Biochemical Recurrence			
No	37	7.62	172.5
Yes	5	20.3	171.5
P		0.24	0.41
Clinical T stage			
T1c*	20	9.47	169.5
T2a-c†	21	9.19	174.1
P		0.24	0.29

* Tumor identified by needle biopsy, but clinically inapparent and not palpable
† Tumor is palpable

Supplementary Table S3. Association between cfDNA and continuous clinical features for patients with localized disease.

Clinical Feature		cfDNA Concentration	Average Fragment Size
Age at Diagnosis (years)			
Number of Patients	45		
Average (SD)	62.7 (7)		
P		0.35	0.60
PSA at Diagnosis (ng/mL)			
Number of Patients	45		
Average (SD)	8.7 (7)		
P		0.86	0.85
Decipher Score			
Number of Patients	12		
Average (SD)	0.66 (0.2)		
P		0.81	0.65
Time to Salvage Therapy (days)			
Number of Patients	39		
Average (SD)	1044 (926)		
P		0.63	0.25
Average Fragment Size (bp)			
Number of Patients	45		
Average (SD)	176 (21)		
P		0.67	-
CAPRA-S Score			
Number of Patients	45		
Average (SD)	3.7 (3)		
P		0.50	0.12
